# Relative validity of a semiquantitative food frequency questionnaire designed for schoolchildren in western Greece

**DOI:** 10.1186/1475-2891-8-8

**Published:** 2009-02-05

**Authors:** Maria Roumelioti, Michalis Leotsinidis

**Affiliations:** 1 Laboratory of Public Health, Medical School, University of Patras, Patras, Greece

## Abstract

**Background:**

The use of food frequency questionnaires (FFQs) has become increasingly important in epidemiologic studies. During the past few decades, a wide variety of nutritional studies have used the semiquantitative FFQ as a tool for assessing and evaluating dietary intake. One of the main concerns in a dietary analysis is the validity of the collected dietary data.

**Methods:**

This paper discusses several methodological and statistical issues related to the validation of a semiquantitative FFQ. This questionnaire was used to assess the nutritional habits of schoolchildren in western Greece. For validation purposes, we selected 200 schoolchildren and contacted their respective parents. We evaluated the relative validity of 400 FFQs (200 children's FFQs and 200 parents' FFQs).

**Results:**

The correlations between the children's and the parents' questionnaire responses showed that the questionnaire we designed was appropriate for fulfilling the purposes of our study and in ranking subjects according to food group intake.

**Conclusion:**

Our study shows that the semiquantitative FFQ provides a reasonably reliable measure of dietary intake and corroborates the relative validity of our questionnaire.

## Background

Validation studies on the use of food frequency questionnaires (FFQs) have generally demonstrated the reliability and suitability of this tool in ranking the nutrient intake of individuals, although there have been a few exceptions [1-4]. However, when dietary data on children is collected, their confidence of response is required. There is a fairly rapid increase in the capability of children to respond to eating behavior inquiries beginning at 7 or 8 years of age. By 10 to 12 years of age, children can provide their own responses. During this period of increase in capability, there is a concomitant need to maintain a level of confidence in the respondent.

A few years ago, we started an extensive research study in an attempt to assess the prevalence of obesity and the nutritional habits of children in southwestern Greece. To carry out this assessment, we created an FFQ that also included questions about physical activities and sedentary behavior. The questionnaire was approved by the Greek minister of education and the Hellenic Data Protection Authority. However, this FFQ had to be evaluated before large-scale use. In this paper, we present the validation methodology we followed to evaluate the relative validity of our newly created FFQ.

## Methods

The validation study addressed children of urban and rural areas of western Greece (three schools from urban areas and three schools from rural areas). The subjects of the study included a sample of 200 children, out of 350 children. The age of the children (boys and girls) was between 10 and 12 years (fourth, fifth, and sixth grades), and the age of their respective parents (200 parents, mostly mothers) was between 28 and 55 years. The data were collected using a semiquantitative FFQ [5] and a 24-hour-recall method for the forthcoming 7 days [6]. At school, children were randomly selected to participate. The children's responses to the semiquantitative FFQ were recorded by the interviewer. Data concerning age, place of residence, weight, height, and lifestyle factors were also recorded. The children's questionnaire contained 65 food items and a list of colour photographs of 2 differently sized portions for 40 typical Greek foods [7]. Each food item was presented proportionally (1/2, 1, 1 1/2 portions). For statistical reasons, the food items were grouped into 12 food sets. The frequency of consumption of any food item was categorized as 1 to 7 days per week, 1 to 2 days per month, or never. Subsequently, we applied reproducibility procedures, validated methodological issues [8-10], and changed the FFQ to avoid inaccurate or random responses. We removed food items that were rarely consumed from the FFQ because we noticed that the children had difficulty remembering their consumption frequency for these food items and would confuse them. We confirmed that the number of food items used in nutritional studies is not necessarily a major factor in reliability measurements. Increasing the number of food items in an FFQ usually corresponds to an increased number of missing values [11,12]. Because we have noticed that factors related to spontaneous physical activities and stress do not have a significant role in the statistical analysis [13], we limited the number of questions related to these factors so as to adhere to the strict time frame (20 minutes) of the questionnaire.

The parents' questionnaire gathered general information (e.g., sex, age, nationality, profession, education, marital status, height, weight) about both parents. The remainder of the parents' questionnaire concerned their children's food consumption. We also added some supplementary questions related to the nutritional habits of the whole family and removed the pictures of food items and portions.

The parents were interviewed by telephone. The duration of each interview was about 20 minutes and usually involved only mothers. To establish an appropriate degree of confidence between the interviewer and the parents, we avoided asking personal questions. After the completion of each questionnaire, the interviewer corrected the collected data, using some cross-check questions usually derived from the answers that were already given.

To accommodate the statistical analysis, we encoded the questionnaire responses (200 children's questionnaires and 200 parents' questionnaires) as follows: The values were normalized and grouped into three subgroups. The first subgroup represented low consumption values (≤ 2 portions), the second subgroup included medium consumption values (3–4 portions), and the third subgroup comprised high consumption values (≥ 5 portions).

### Statistical analysis

To assess the validity of the FFQ, statistical analysis was carried out using Statistical Product and Service Solutions (SPSS) version 15.0. Spearman product-moment correlation coefficients were used to compare the children's and the parents' FFQ responses, and statistical significance was set at *P *< 0.05. Furthermore, because assessing association was not our only aim (and this was not a hypothesis testing problem), statistical tests of agreement were applied to the data collected to assess the strength of agreement between the responses of the two sets of questionnaires. For this measurement of agreement, we used the kappa measure. The responses were calculated by cross tabulation of the data, and the agreement analysis was interpreted in accordance with the guidelines shown in Table 1 (slightly adapted from Landis and Koch, 1977) [14].

**Table 1:** Agreement analysis guidelines

**Value of kappa**	**Strength of agreement**
*< 0.20*	Poor
*0.21–0.40*	Fair
*0.41–0.60*	Moderate
*0.61–0.80*	Good
*0.81–1.00*	Very good

## Results

A total of 200 students and 200 parents from western Greece participated in this study, with the majority of respondents living in urban areas (79% from urban areas vs. 21% from rural areas). The children's sample consisted of 47% boys and 53% girls. A much higher percentage of women (97%) comprised the parents' sample than men (3%). The sample comprised 98.5% Greeks and 1.5% foreigners. The education level of the women respondents was as follows: elementary: 27.8%, secondary: 43.3%, and college/higher education: 28.9%. In comparison, 33.3% of the men respondents had achieved a secondary education level and 66.7% had attained a college/higher education level. Finally the marital status of the sample was as follows: 95.5% married, 4%, divorced, and 0.5% single.

To measure the degree of "straight-line" association between the values of the responses from the two sets of questionnaires, we used the correlation coefficient as the method of analysis. As shown in Tables 2 and 3, a very strong correlation exists between the responses of the children and their parents in the food groups and food items as well as the physical activities, sedentary behavior and social status (where the value of *r *is >0.69). The values of *r *for the rest of the food groups indicate that a moderate association exists between the responses of the subjects.

**Table 2:** Correlation coefficient for validity of the Food frequency questionnaire

**Food items/groups**	***r***
***Cornflakes***	0.58
***Milk chocolate***	0.61
***Fruit***	0.63
***Pasta***	0.66
***Rice***	0.66
***Delicatessen***	0.66
***Potatoes***	0.67
***Sweets***	0.67
***Boiled vegetables***	0.69
***Fruit juices***	0.70
***Souvlaki***	0.70
***Breakfast***	0.71
***Pizza***	0.72
***Meat***	0.72
***Biscuits***	0.73
***Egg***	0.74
***Fresh vegetables***	0.76
***Legumes***	0.76
***Bread***	0.77
***Sandwiches***	0.77
***Fish food***	0.78
***Potato chips***	0.79
***Refreshments***	0.79
***Pies***	0.82
***Dairy***	0.84
***Milk***	0.90

**Table 3:** Correlation coefficient for validity of physical activities, sedentary behaviour, and social status

**Physical activities/social status**	***r***
***Music lessons***	0.96
***Marital status***	0.95
***Sports***	0.91
***Dancing***	0.90
***Father's education level***	0.88
***Mother's education level***	0.88
***Studying***	0.88
***TV***	0.69
***Video games***	0.53
***Sleeping***	0.43

To assess the validity of the FFQ, we carried out tests of agreement between responses received from the children's FFQ and the parents' FFQ, which were calculated by cross tabulation of the data. As we can see in Table 4, agreement analysis results revealed very good agreement for the food item (i) milk and good agreement for the food groups (i) fish food, (ii) pies, (iii) delicatessen, and (iv) dairy and for the food items (i) cheese, (ii) legumes, and (iii) pizza. Fair agreement between the two sets of questionnaires was determined for the food groups (i) pasta, (ii) boiled vegetables, (iii) refreshments, (iv) fruit juices, and (v) fresh vegetables and for the food items (i) rice, (ii) biscuits, (iii) potato chips, (iv) souvlaki, (v) egg, (vi) milk chocolate, (vii) bread, and (viii) meat. Fair agreement between the two sets of questionnaires was also ascertained for breakfast. Finally, the statistical analysis showed that fruit, sweets, sandwiches, and potatoes had poor agreement.

**Table 4:** Agreement for validity of the FFQ

**Food items/groups**	**Value of kappa**	**Strength of agreement**
***Potatoes***	0.361	Poor
***Fruit***	0.381	Poor
***Sweets***	0.400	Poor
***Sandwiches***	0.403	Poor
***Fresh vegetables***	0.460	Fair
***Meat***	0.473	Fair
***Cornflakes***	0.477	Fair
***Fruit juices***	0.481	Fair
***Breakfast^1^***	0.483	Fair
***Bread***	0.502	Fair
***Milk chocolate***	0.525	Fair
***Egg***	0.537	Fair
***Souvlaki***	0.540	Fair
***Refreshments***	0.549	Fair
***Potato chips***	0.569	Fair
***Boiled vegetables***	0.574	Fair
***Pasta***	0.583	Fair
***Biscuits***	0.588	Fair
***Rice***	0.592	Fair
***Pizza***	0.594	Good
***Dairy***	0.605	Good
***Sausages***	0.626	Good
***Pies***	0.657	Good
***Fish food***	0.715	Good
***Legumes***	0.725	Good
***Cheese***	0.731	Good
***Milk***	0.812	Very Good

^1 ^Breakfast was assessed on a yes or no basis. Refreshments were measured using the can-base unit of commercial refreshments.

## Discussion

In the present investigation, we evaluated the validity of using a 200-item semiquantitative FFQ in a large prospective study in western Greece. An important consideration when interpreting the results is the fact that a great percentage of the food groups and food items that were statistically compared confirmed the validity of our questionnaire. To assess the relative validity, we chose the 24-hour-recall method for the forthcoming 7 days as the reference method. Because the interviews with the children were conducted at school, and the phone interviews were carried out with the parents after they had been previously notified and interview times had been set, the response rate was high. Only two of the parents refused to participate because of time limitations. Taking into consideration the age group of our sample, as well as the fact that children in this age group have not completely developed the ability to think abstractly and calculate their average food intake [15], we interviewed fourth- and fifth-grade children with great attention. To avoid errors in estimation of the children's food consumption, the interviewer used photographs of food portions and tried to clarify the real food intake. Special attention was given to determine the maturity level of each child and its effect on the child's answers.

The eating patterns selected for this study and the methodological approaches chosen to analyze the dietary data were based on an extensive review of the literature [16-20], food consumption patterns [21,22], total gram amount of food/beverages consumed by meal, total number of snack periods, and total eating episodes [23-25]. A comparison of the results revealed very good agreement for milk. Good agreement was determined for several food groups (fish food, pies, delicatessen, and dairy), as well as for several food items (cheese, legumes, and pizza). The children's and parents' questionnaire responses relating to milk consumption were in almost perfect agreement – most likely because milk is a daily source of nutrients in childhood and is consumed at standard time frames within a day (e.g., morning, afternoon, and evening). Consequently, it was easier to calculate the frequency and the quantity of milk consumption. Accordingly, we could explain the good agreement found for several food groups (fish food, pies, delicatessen, and dairy) and for several food items (cheese, legumes, and pizza). It is well known that Greek nutritional culture primarily consists of fish food, dairy, cheese, and legumes [26]. We noticed that the children and their parents reported the same frequencies for legumes and fish food because most Greeks have standard days for the consumption of these food items. Legumes are usually consumed on Wednesdays and Fridays because of fasting, whereas fish is traditionally consumed on Saturdays. Based on these findings, we concluded that the strong agreement found for some food items was related to the traditional weekly nutrition routine of Greeks. The calculation of frequency consumption revealed high accuracy.

Of particular interest was the poor agreement between the children's and the parents' questionnaire responses regarding the consumption of several food items – specifically, fruit, sweets, sandwiches, and potatoes. The statistical analysis showed that although poor agreement was attained for these food items, the parents had occasionally underestimated the children's consumption of sweets, sandwiches, and potatoes, and overestimated the consumption of fruit. In our opinion, the parents' overestimation/underestimation of their children's consumption of certain foods may be related to the fact that parents may not (consciously or subconsciously) want to divulge their children's nutritional choices. Their responses, which suggested that their children possess a balanced diet) may be based on wishful thinking. We suspect that some parents had a tendency to overlook the quantity and the frequency of many food items that their children consumed.

Based on our findings, we feel confident in the appropriateness of this questionnaire in examining the food consumption of the Greek student population and in ranking the subjects according to food group intake. However, findings of similar studies have shown that dietary intake cannot be estimated without the possibility of error and that great attention should be given when selecting and employing the most appropriate dietary data collection method as well as the analytical and statistical methods [27-32].

## Conclusion

Our study shows that the semiquantitative FFQ provides a reasonably reliable measure of dietary intake and corroborates the relative validity of our questionnaire. The relative validity of FFQs that calculate a limited number of food items is generally more effective than the relative validity of questionnaires that are based on overall food consumption [33-35]. Questions that arise are mainly related to the nature of errors (bias or random), the validation of the food consumption, and the use of appropriate statistical methods. However, it is a robust methodology and can be applied in a large-scale epidemiologic study.

## Competing interests

The authors declare that they have no competing interests.

## Authors' contributions

MR participated in data collection, data analysis and manuscript development. ML participated in data analysis and manuscript editing.
